# Machine learning modeling of predictive external corrosion rates of spent nuclear fuel carbon steel canister in soil

**DOI:** 10.1038/s41598-022-24783-5

**Published:** 2022-11-24

**Authors:** Thuy Chung Nguyen, Yoon-Sik So, Jin-Soek Yoo, Jung-Gu Kim

**Affiliations:** School of Advanced Materials Science and Engineering, Sungkyunkwan University, 2066, Seobu-ro, Jangan-gu, Suwon, Gyeonggi-do 440-746 Republic of Korea

**Keywords:** Energy science and technology, Engineering, Materials science, Mathematics and computing

## Abstract

Soil corrosion is always a critical concern to corrosion engineering because of the economic influence of soil infrastructures as has been and has recently been the focus of spent nuclear fuel canisters. Besides corrosion protection, the corrosion prediction of the canister is also important. Advanced knowledge of the corrosion rate of spent nuclear fuel canister material in a particular environment can be extremely helpful in choosing the best protection method. Applying machine learning (ML) to corrosion rate prediction solves all the challenges because of the number of variables affecting soil corrosion. In this study, several algorithms of ML, including series individual, boosting, bagging artificial neural network (ANN), series individual, boosting, bagging Chi-squared automatic interaction detection (CHAID) tree decision, linear regression (LR) and an ensemble learning (EL) merge the best option that collects from 3 algorithm methods above. From the performance of each model to find the model with the highest accuracy is the ensemble stacking method. Mean absolute error performance matrices are shown in Fig. 15. Besides applying ML, the significance of the input variables was also determined through sensitivity analysis using the feature importance criterion, and the carbon steel corrosion rate is the most sensitive to temperature and chloride.

## Introduction

Soil corrosion has received much attention because there are many instances of underground infrastructure such as spent nuclear fuel canisters containing nuclear waste^1–4^. This infrastructure is essential and plays an important role in modern life. The long-term preservation of radioactive waste remains a major challenge worldwide. Fuel in these systems and those that will be discharged may need to be stored for periods of up to 100 years. There are many types of canisters used for underground waste storage such as carbon steel, stainless steel, nickel alloys, and titanium alloys… if the canister is corroded, cracked, and cannot be replaced after a long time, it will have a significant economic impact^5^. Knowing the corrosion rate of metal and the properties of the soil in advance is very helpful for engineers to find suitable protection methods for the pipeline^6–9^. However, predicting the corrosion rate in complex environments such as soil is not easy because the soil environment has many factors that affect the corrosion rate including the chemical concentration in the soil water, soil moisture, and soil structure^10^. Matteo Stefanoni et al. have published a very successful study describing an equation that can predict the corrosion rate as a function of porosity in which water fills the voids in the soil^11,12^. Mohamed El Amine Ben Seghier et al. predicted the internal corrosion rate of oil and gas pipelines^13^. However, no researchers have studied the factors that affect the external corrosion rate of underground metals based on the composition of the soil solution and its temperature.

In the modern world, almost all manual tasks can be automated using machine learning algorithms^14^. Machine learning (ML) has a wide range of potential industrial applications^15,16^. The machine learning method is suitable for predictive models with several variables^17^. Recently, many scientific fields applied machine learning to multidisciplinary prediction^17–19^. Even in the field of corrosion, many scientists have applied machine learning to predict the corrosion rate in the atmosphere, the performance of corrosion inhibitors, and corrosion behavior^20–24^. However, there are not many studies that focus on predicting the corrosion rate of carbon steel canisters in the soil environment. Our previous studies have involved predicting the corrosion rate of carbon steel based on the influence of pH, chloride, and sulfate concentration of soil solution, using a response surface method (RSM)^10^ and pH, chloride, temperature of soil solution with different investigated range values using RSM and an artificial neural network (ANN)^25^. The limitation of our previous studies is that there are only three corrosive factors.

Therefore, this study aims to predict the corrosion rate of carbon steel, a material used as a cost-effective canister in a soil environment with a full range of factors that consider corrosion under real soil conditions using several machine learning algorithms. Specifically, this work finalizes the prediction of soil corrosion of carbon steel canister and demonstrate how to apply machine learning to corrosion science. Three ML algorithms and ensemble learning methods were employed to predict the corrosion rate focusing on components of the soil solution and its temperature using IBM SPSS modeler software. They are optimized to find the model with the best parameters to predict corrosion rate. IBM SPSS modelers are powerful support software to make machine learning easier and more accessible to non-data scientists. Five factors affecting the corrosion rate were selected in this study including pH, chloride, bisulfide, sulfate, and temperature. This study also looks for the most sensitive factors affecting external corrosion in soil.

## Methodology

Machine learning (ML) is a type of artificial intelligence (AI) that enables software applications to become more accurately predict outcomes without being explicitly programmed^26,27^. ML algorithms give us historical data as inputs to predict new output values. ML is important because it provides people with a view of trends in things, events, and even human behavior^28^. Many of today’s leading companies are making ML a central part of their operations, and ML has become a significant competitive differentiator for many companies^29^. ML also has its limitations but there are already practical applications where machine learning does a great job, such as image processing, text analysis, data mining (DM), video games, and robotics. The ML approach is essential for the development of corrosion science. This study determines the problem prediction of the external corrosion rate of carbon steel canisters in soil and selects an optimal and suitable ML algorithm.

### Selection of single algorithm machine learning method

**Figure 1:**
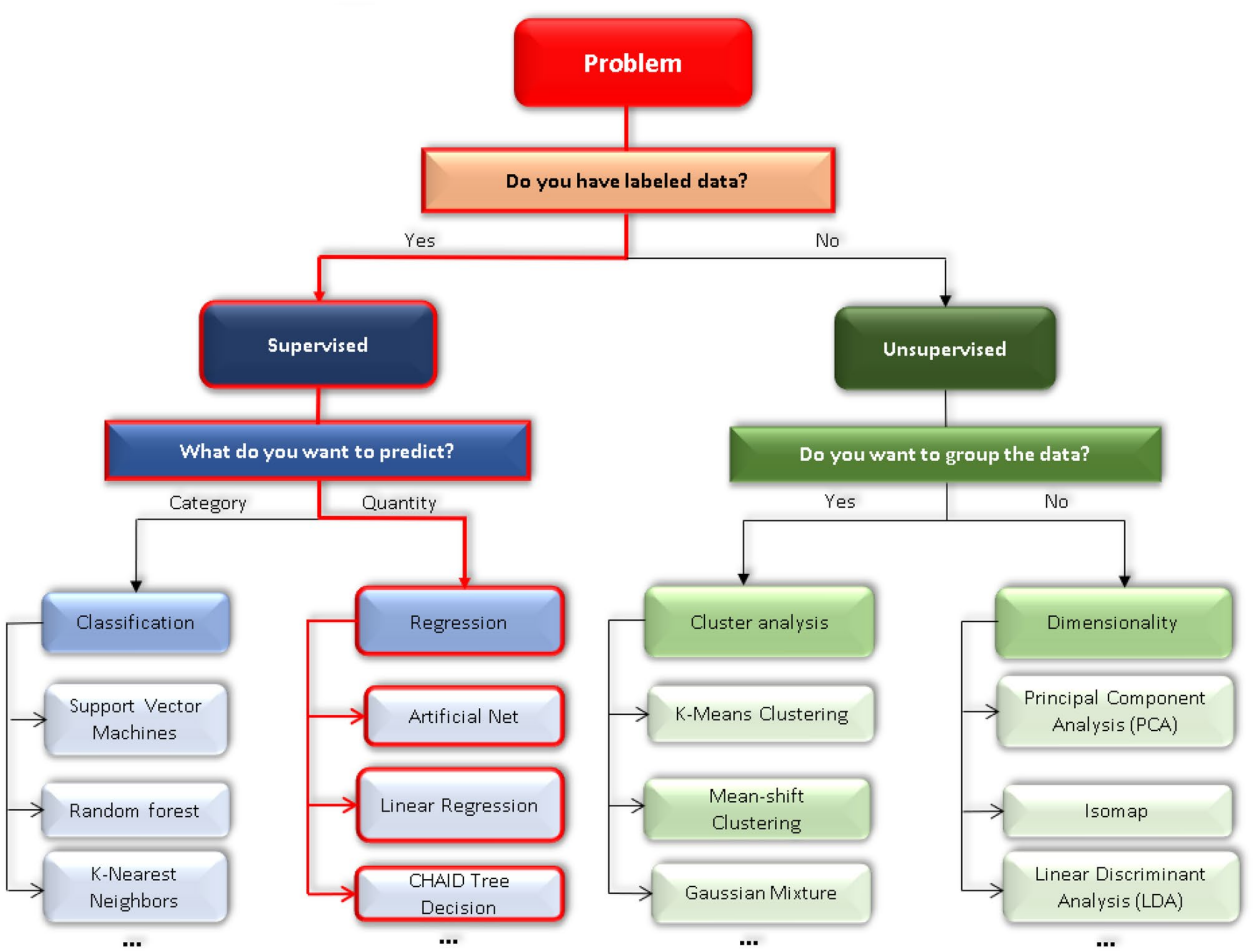
Various categories of machine learning algorithms.

The library of ML algorithms is very diverse^30,31^. Therefore, identifying a suitable algorithm is a key issue in this study. Figure 1 summarizes how we chose the algorithm for this study.

There are two types of popular ML algorithms including supervised and unsupervised learning^32^. Supervised learning is an algorithm that predicts the output of new data based on known pairs of inputs and outcomes. This pair of data is also known as data and label. In addition to building strong models, collecting, and labeling reasonable data also play a key role to solve problems in supervised learning. Meanwhile, unsupervised learning is a group of algorithms that allow the machine to learn on its own and find a certain pattern or configuration hidden in an unlabeled dataset. This means that it only has the input dataset and has no idea what the outcome is. In other words, using these methods is more about characterizing the data^32^. Supervised learning has higher accuracy than unsupervised learning, and the data of this study are labeled since it has a specific predictor value and a specific soil corrosion current density^27^. Therefore, the supervised learning algorithm is completely feasible for this study.

The supervised algorithm is further subdivided into two main parts: classification and regression. The most important difference between regression and classification algorithms is that a classification algorithm is often used to predict categories and discrete values, while regression algorithms are often used to predict continuous values. In this study, the corrosion current density is a specific quantity, therefore the regression method was chosen.

There are many other algorithms of regression supervised learning—so which algorithm will be best suited to make predictions in this study? In the field of ML, there is a no free lunch (NFL) theorem: “All optimization algorithms perform equally well when their performance is averaged across all possible problems”. In short, this assumes that there is no single best optimization algorithm for all predictive modeling problems^33^. For example, you cannot say that an artificial neural network is always better than a decision tree or vice versa because there are many influencing factors such as the size and orthogonality of the dataset. Therefore, in this study, several popular machine learning algorithms were implemented for the prediction of soil corrosion rate problems so that corrosion scientists can understand the implementation of each type and know how to find the optimal prediction algorithm. In this study, we focused on the three single regression supervised learning ML algorithms: artificial neural network (ANN), Chi-squared automatic interaction detection (CHAID) tree decision, and linear regression (LR). In addition to the selected single algorithms, the ensemble learning (EL) method was applied in this study to increase predictive performance. The EL method is an algorithm that merges several algorithms to obtain better predictive performance than single algorithms. A more detailed description of the ensemble algorithm will be described in the next section.

### Ensemble learning

**Figure 2:**
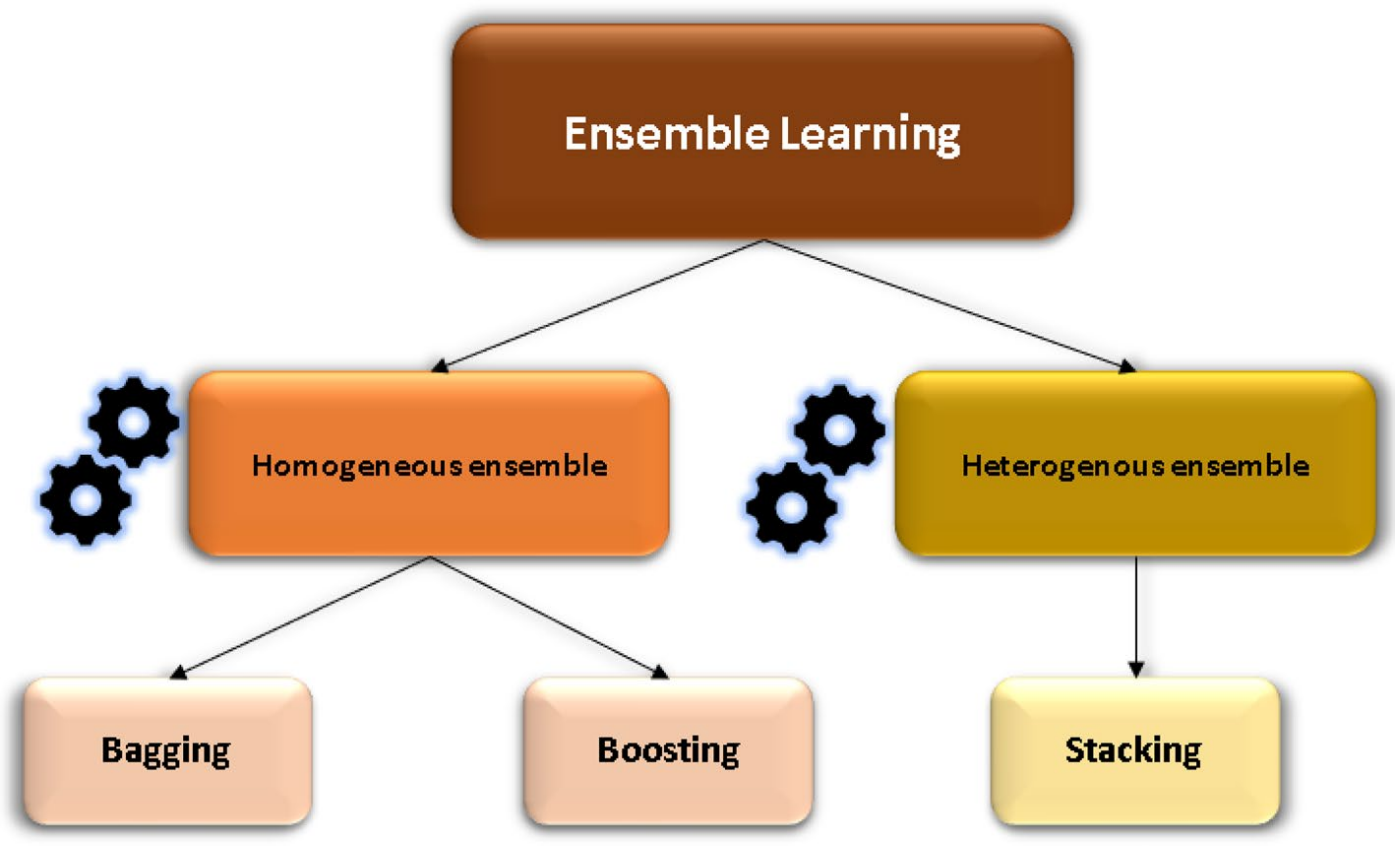
Classification of ensemble learning methodologies.

The ensemble learning method is an idea of combining different models, which can do a better job of different types of work. Properly combined models form a powerful hybrid model that can improve the overall performance compared to using the model alone^34^. Figure 2 simply depicts EL methodologies.

There are two types of EL methods: homogeneous EL methods and heterogeneous EL methods. The homogeneous ensemble method is a method of building an ML model many times with the same amount of training data. The second is the heterogeneous ensemble method, which is a method of building a model using different ML algorithms, and each algorithm will use the same amount of training data. There are two types of ensemble homogeneous methods, bagging and boosting. Both methods (homogeneous and heterogeneous) will be applied to improve the accuracy of prediction in the study, and the two types of ensemble methods will be described in detail in the next section.

#### Homogeneous ensemble method

**Figure 3:**
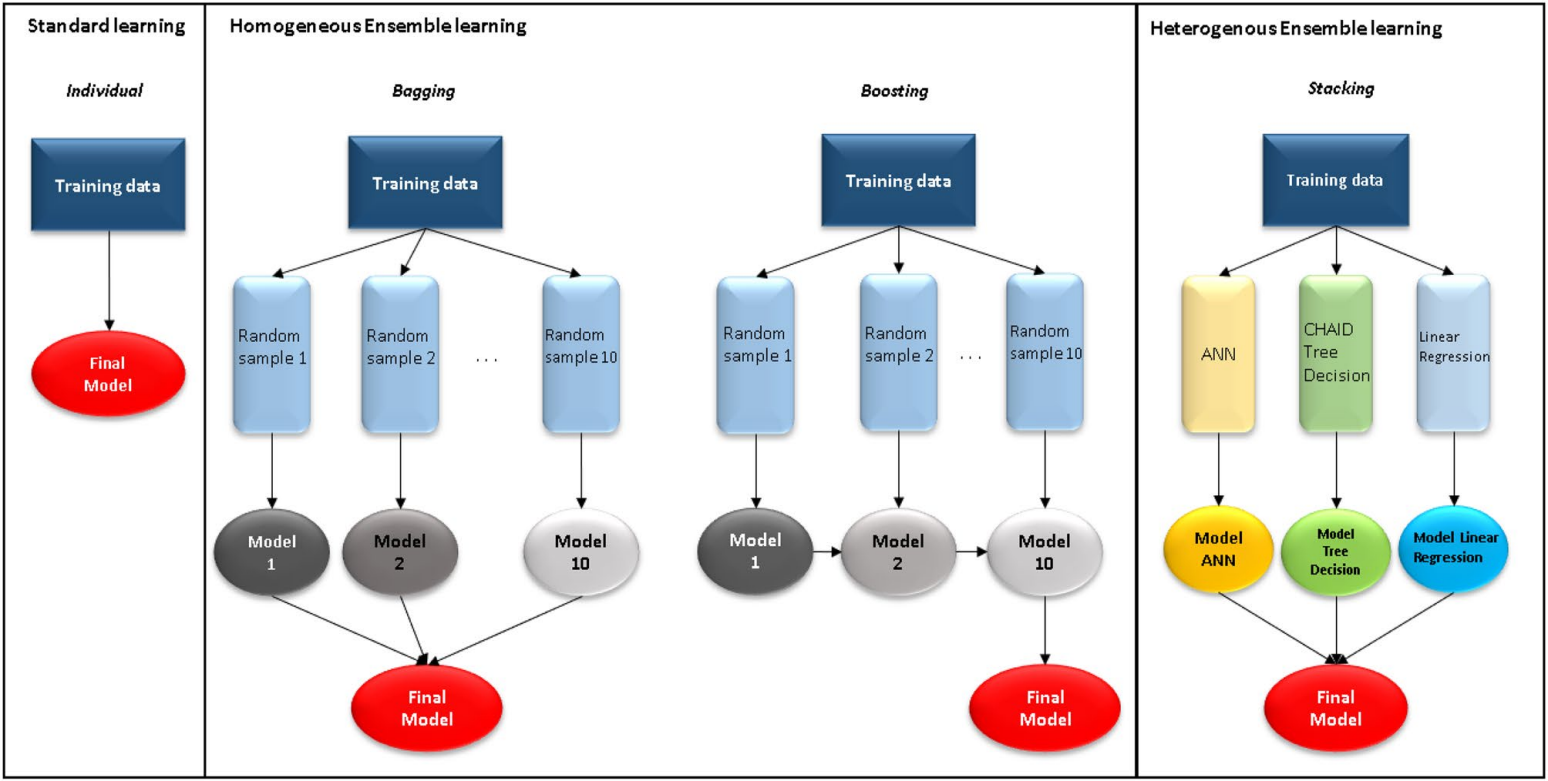
The architecture of standard learning, homogeneous ensemble learning, heterogeneous ensemble learning.

As shown in Fig. 3, homogeneous ensemble methods can be divided into two types: parallel ensemble methods (bagging) and sequential ensembles (boosting). A problem when running the prediction will be overfitting due to high variance or underfitting by an excessively high bias. To solve that problem, we considered decreasing variance and decreasing bias. In the homogeneous ensemble learning method, bagging will help reduce variance, and boosting will reduce bias. If the single algorithms method will use the training dataset to run and gain the best fit model, the homogeneous ensemble methods (bagging and boosting) will use random training data set to run many times to get an output with a different number of models. In bagging, the weak models will be trained independently in parallel, but in boosting, they will be trained sequentially. Thus, a sequence of models is built, and the weight of the data that was wrong in the previous model increases with each new model repeated. This weight reallocation helps the algorithm determine the parameters it needs to improve its performance. In this study, standard learning, and homogeneous ensemble learning (bagging, boosting) were used in artificial neural networks. Ten component models were used for boosting or bagging.

#### Heterogeneous ensemble method

As shown in Fig. 3, the heterogeneous ensemble method uses different base algorithms to ensure the diversity of the set, in contrast to the homogeneous ensemble. In this study, after running standard learning, bagging, and boosting methods for each algorithm, the model with the best results of ANN, CHAID, and linear regression was selected forward to run the stacking method to increase the predictive performance from the three algorithms above.

## IBM SPSS modeler and results

**Figure 4:**
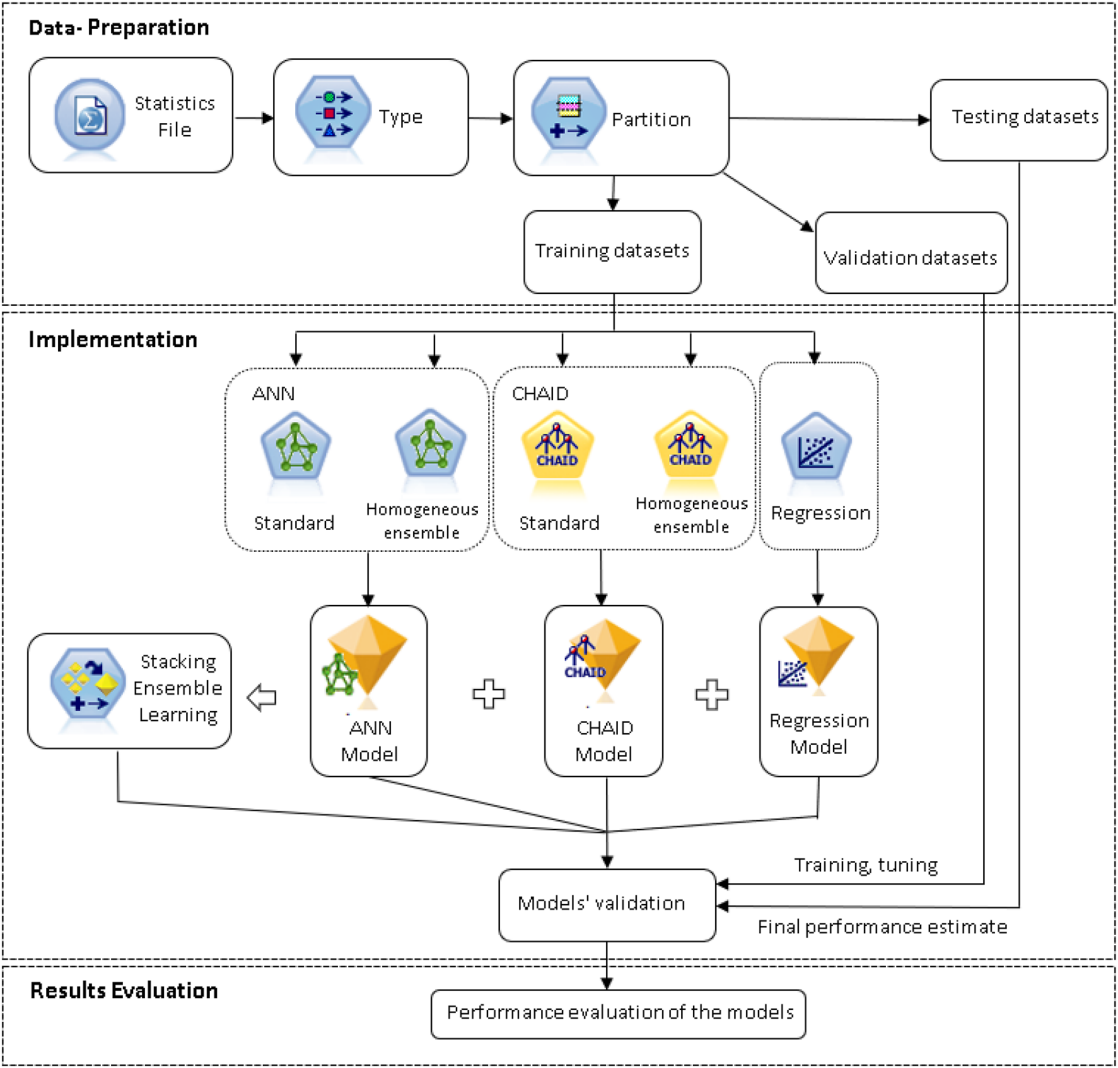
Steps performed on IBM SPSS modelers for the prediction of the external corrosion rate of carbon steel in soil.

IBM SPSS Modeler is a leading visual data science and machine learning solution. This enables users to mine data and modern applications with complete algorithms and models ready to use immediately. Figure 4 shows the steps performed on IBM SPSS modelers and how each step is performed, and the result will be explained in the next sections.

### Data-preparation

Data preparation consists of three steps which are to collect data, classify data, and divide the dataset for training, testing, and validation. To collect the data, the purpose of the study must be defined. In this study, the objective is to predict the corrosion rate, and predictors will be the factors affecting underground corrosion. Table 1 summarizes the predictors, targets, and investigated range of predictors in this study. Although many factors affect soil corrosion, it is challenging to know how many factors affect corrosion in soil. A reasonable number of factors in this stub study were chosen because they are of interest to affect corrosion and they are easy to tune to run the experiment.

**Table 1:** Defined predictors and targets to predict the corrosion rate of carbon steel in soil.

Roles	Investigate Factor	Units	Range value
Predictors	pH, X_1_		4–10
	Chloride, X_2_	ppm	35.5–3550
	Bisulfide, X_3_	ppm	70.04–33,000
	Sulfate, X_4_	ppm	0–670.04
	Temperature, X_5_	°C	20–80
Target	Corrosion current density, Y	μA/cm^2^	4.2–46.7

**Figure 5:**
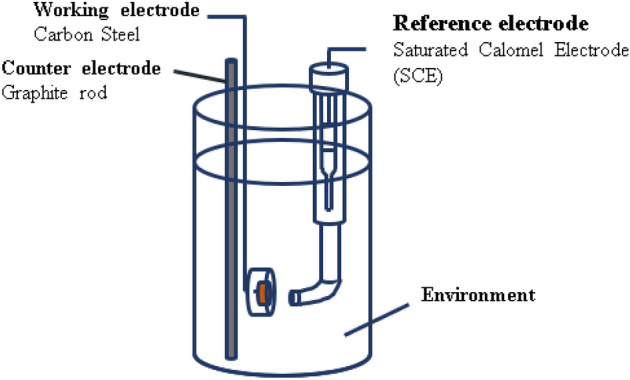
Three-electrode setup for collecting data.

In AI, more data is always better, because more data results in additional training and a smarter model. If the data are well prepared according to a basic data prep checklist, they will be ready for machine learning, and accurate results will be obtained. In this study, some data were collected from previous studies, and we supplemented the data by running electrochemical experiments in Fig. 5, which are quite sufficient for an accurate result.

**Figure 6:**
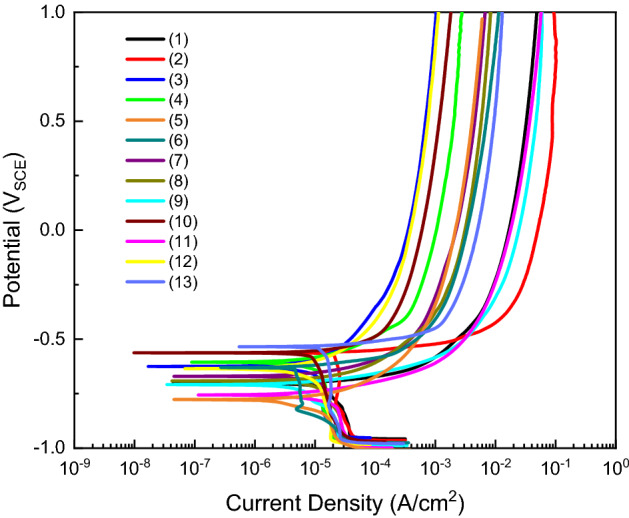
Potentiodynamic polarization curves of carbon steel with variation in pH, chloride, and bisulfide.

Carbon steel SPW400 was used as the working electrode with a composition of 0.04 wt.% S, 0.04 wt.% P, 0.25 wt.% C, and balance Fe (Korean Standard). This material is commonly used in soil industries. The test environment is deionized water with variations in chemical composition and pH. Chloride, bisulfide, and pH adjusted for NaOH, and borate acid saturates, NaCl and Na_2_S were used as listed in Table 1. Temperature changes were controlled using a heating plate. We used a cell consisting of carbon steel SPW400 as the working electrode, a saturated calomel electrode as the reference electrode, and two pure graphites as the counter electrode. Samples were polished with SiC from using 200–600 grit sizes, and the surface was covered with silicone paste to reveal 1 cm^2^ carbon steel. After the sample was dry, the experiment was run in OCP for 3 h and was then potentiodynamically run from − 0.25 vs. OCP to 1 vs. OCP with a scan rate of 0.166 mV/s. After the experiment, the potentiodynamic polarization curve was obtained in Fig. 6 and it was used with the Tafel method to find the corrosion current density value of each experiment. All collected data are given in Table 2, experiments 1–13 were run in this study and experiments 14–43 were collected from our previous studies^10,25^.

**Table 2:** Corrosion current density is affected by pH, chloride, bisulfide, sulfate, and temperature.

Type	Predictors					Target	References
STT	pH	Chloride (ppm)	Bisulfide (ppm)	Sulfate (ppm)	Temperature (°C)	EXP (μA/cm^2^)	
1	8	3550	3300	0	20	24.85	
2	10	3550	3300	0	20	26.55	
3	8	35.5	3300	0	20	6.94	
4	10	35.5	3300	0	20	10.52	
5	8	355	33,000	0	20	14.37	
6	10	355	33,000	0	20	15.51	
7	8	355	330	0	20	14.43	
8	10	355	330	0	20	15.14	
9	9	3550	33,000	0	20	25.15	
10	9	35.5	33,000	0	20	9.48	
11	9	3550	330	0	20	24.95	
12	9	35.5	330	0	20	7.7	
13	9	35.5	3300	0	20	16.4	
14	6	85.2	70.04	70.04	20	4.2	^10^
15	4	85.2	370.04	370.04	20	4.9	
16	8	85.2	370.04	370.04	20	4.8	
17	6	85.2	670.04	670.04	20	5.0	
18	4	85.2	70.04	70.04	20	5.4	
19	8	585.2	70.04	70.04	20	5.5	
20	6	585.2	370.04	370.04	20	5.7	
21	6	585.2	370.04	370.04	20	5.8	
22	6	585.2	670.04	370.04	20	5.6	
23	4	1085.2	670.04	670.04	20	6.2	
24	8	1085.2	70.04	670.04	20	6.3	
25	6	1085.2	370.04	70.04	20	8.6	
26	4	1085.2	370.04	370.04	20	9.1	
27	8	1085.2	670.04	370.04	20	9.2	
28	6	585.2	0	670.04	20	9.4	
29	8.5	35.5	0	0	20	4.2	^25^
30	10	355	0	0	20	10.3	
31	7	355	0	0	20	15	
32	8.5	3550	0	0	20	15.5	
33	10	35.5	0	0	50	16.4	
34	7	35.5	0	0	50	19.4	
35	8.5	35.5	0	0	80	19.9	
36	8.5	355	0	0	50	26.9	
37	8.5	355	0	0	50	26.8	
38	8.5	355	0	0	50	26.7	
39	10	3550	0	0	50	28.4	
40	10	355	0	0	80	31.5	
41	7	355	0	0	80	35.1	
42	7	3550	0	0	50	43.7	
43	8.5	3550	0	0	80	46.7	

After collecting and classifying factors, the datasets were split into the training set, testing set, and validation set in the partition step. The training set is the data set used to train the model. The algorithms will learn the models from this training set. The validation set was created to periodically evaluate the trained model. The model after training will adjust the parameter based on the results of the regular evaluation of the validation set. To know if an algorithm or model is good or not, the model needs to be evaluated after being trained through a test data set, also known as a test set. In general, validation data typically helps tune the algorithms, and testing data provides the final assessment. In this study, 70% of the dataset was used as the training set, 15% of the dataset was used as the testing set, and 15% of the data set was used as the validation set.

### Implementation and evaluation

The selected algorithms (ANN, CHAID Tree Decision, Linear Regression, Stacking Ensemble) were carried out after the data preparation step. A detailed description of each single algorithm ensemble learning method results is provided below.

#### Artificial neural network (ANN) algorithms

ANNs are mathematical models built through biological neurons. ANNs consist of groups of jobs, and artificial neurons that can connect and process information by passing along the connections and then calculating new values at the nodes. Many ANNs are also tools for modeling nonlinear statistical data.

The two main types of ANN architectures are feed-forward and feedback networks. In feed-forward, signals only flow in the neural network in one direction, whereas back-forward can be repeated. Feedforward is less computationally complicated and is considered less accurate than feedback networks. The traditional feed-forward network is suitable for modeling input data relationships with one or more output responses, especially with soil^35,36^.

Network architecture contains the following three layers: the input layer, the hidden layer, and the output layer. After selecting the type of network architecture, the number of hidden layers and units in each layer must be determined. In this study, the input layer has five units of five factors (temperature, chloride, sulfate, bisulfide, pH), and the output layer has one unit for predicting the corrosion current density of carbon steel. In this study, we chose one hidden layer since this is sufficient for most problems. The number of units for the hidden layer can vary, and there are some empirically based rules, the usual is based on “the optimal size of the hidden layer is usually between the size of the input and the size of the output”^37^. In this study, the size of the input is five and the output is one, to determine the best model, the number of hidden layer units was tested from one to five.

**Figure 7:**
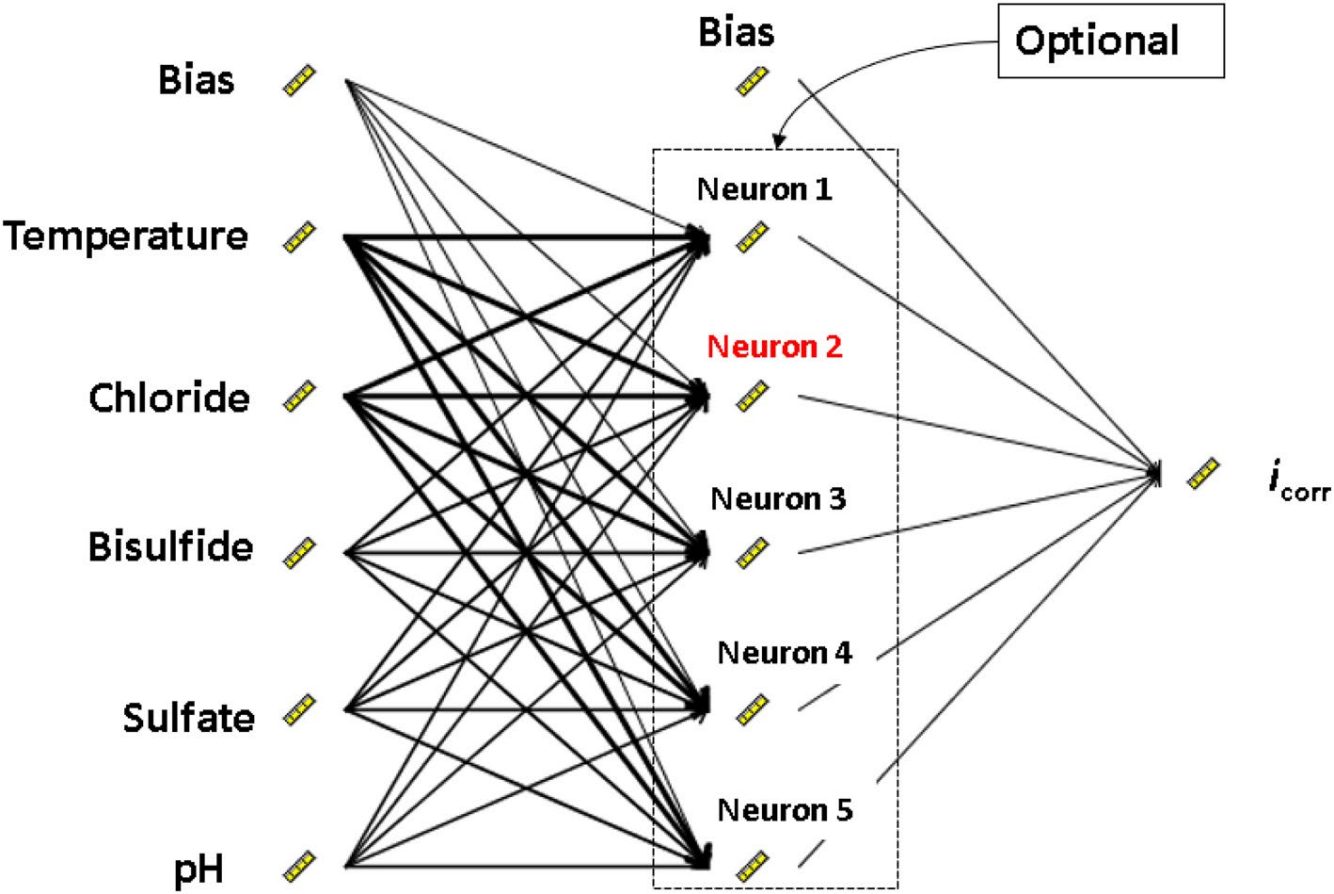
ANN architecture for predicting external corrosion current density of carbon steel in soil.

After building the complete network architecture shown in Fig. 7, the weight and thresholds of all neurons must be determined. Each node *x*_*i*_ in the input layer is connected to each node in the hidden layer *H*_j_*.* Each of those connections is assigned some weights, *w*_ij_*.* At each node in the hidden layer, the total weights of the nodes from the input layer were calculated as ${F}_{j}=\sum_{i}{w}_{ij}{x}_{i}$. The *F*_j_ value was transformed via an activation function, such as a sigmoidal function. This process was repeated on all layers and adjusts the connection weights between the nodes until the mean squared error was minimal and the output layer was reached. Backpropagation, Levenberg–Marquarts, and the conjugate gradient method were the three forms of learning algorithms. A good example of an algorithm is backpropagation (BP), which is the method used in this study.

**Figure 8:**
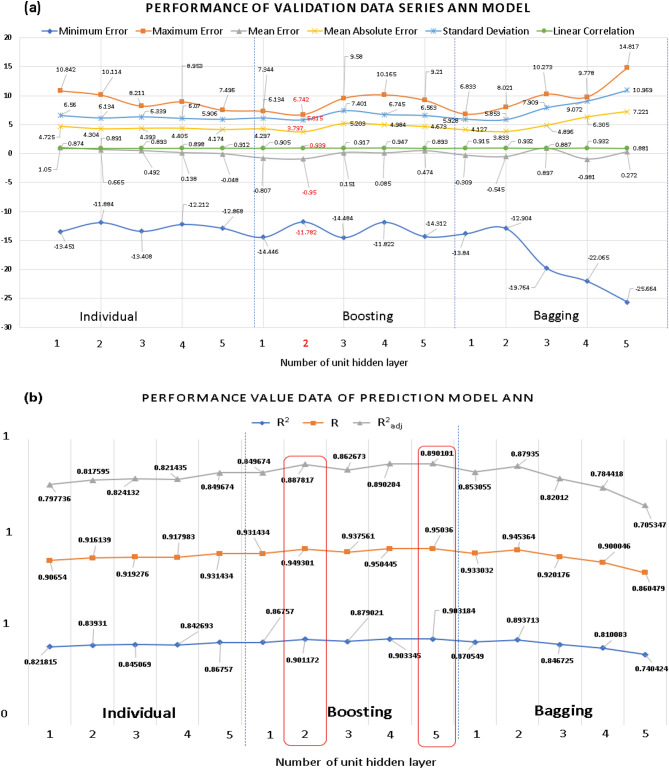
Performance evaluation and comparison (**a**) validation data (**b**) prediction model the single, boosting and bagging models of ANN for modeling *i*_corr_ of carbon steel in soil with variations in the unit of hidden layer.

Of the three methods of the standard and homogeneous ensemble (bagging and boosting) of the ANN algorithm in SPSS IBM modelers with a change in the number of units in the hidden layer from one to five, ANN boosting with two units in the hidden layer was performed with the highest accuracy. The minimum error, maximum error, mean error, mean absolute error (MAE), standard deviation, and linear correlation value was used to evaluate the accuracy of the validation data in Fig. 8a.

From the accuracy-test value of each number of units, we see that 2-unit in the boosting learning method is the best value in the validation data test. This is a good result for prediction. Therefore, two units for the hidden layer and boosting learning method were chosen for predicting the corrosion rate. However, in Fig. 8b when evaluating the R, R^2^, R^2^_adjusted_ values, the 2 units of the hidden layer are not the highest value, but 5 units of hidden layer. That’s not surprising because maybe in 5-units in hidden layer model training data with a close approximation to experimental observations might be better than 2 units hidden layer. When giving validation data to check the accuracy model, the 2 units of the hidden layer prevails. And of course, the 2-unit of hidden is still chosen as the best method because the validation data is the amount of data that is not in the training process. And it is the thing that can evaluate the performance of the model.

**Figure 9:**
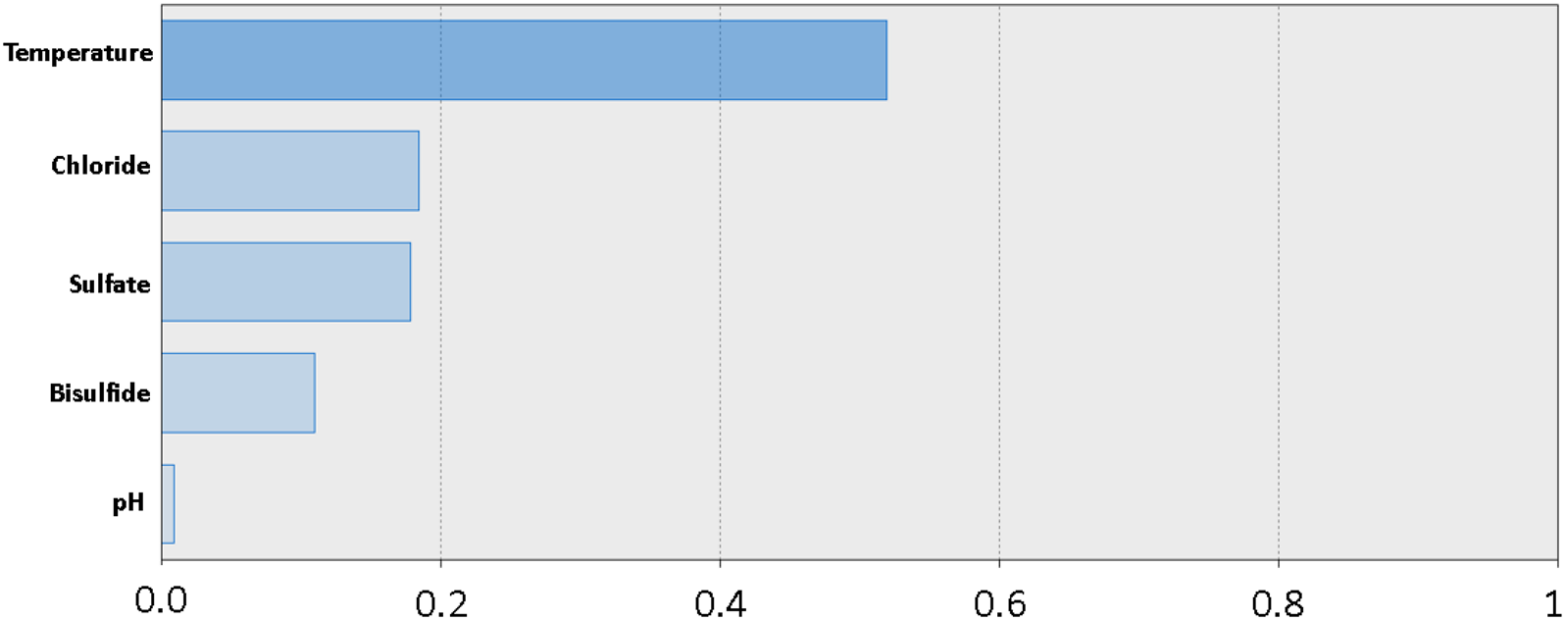
Chart for the standardized effects of temperature, chloride, sulfate, bisulfide, and pH, as predicted by ANN with two units in hidden layer on the corrosion current density of carbon steel in soil.

In analyzing the sensitivity of the factors in the study affecting the corrosion rate using the model ANN with two units in hidden layer, it can be seen that the corrosion rate is the most sensitive to temperature, chloride, and sulfate which have a high influence and bisulfate and pH seem to have a very low effect as shown in Fig. 9^10,38–40^.

#### Decision tree algorithms

The second algorithm chosen in this study is the decision tree. Decision tree learning is one of the earliest and most prominent machine learning algorithms. Decision trees use tree structures to predict the value of an outcome variable. The output of a decision tree is extremely simple to grasp, especially for people lacking an analytical background as they do not require any statistical knowledge to read and interpret them.

**Figure 10:**
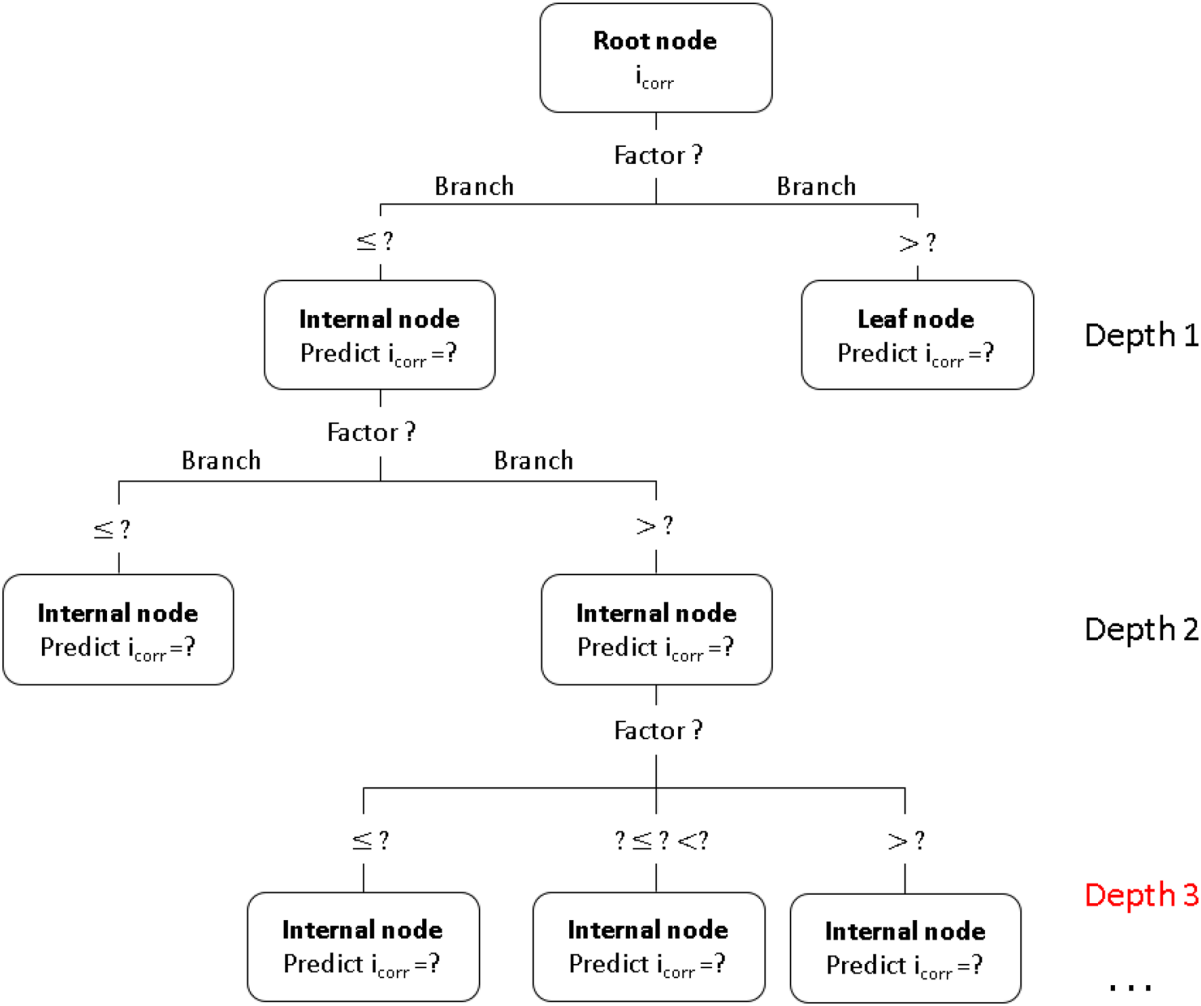
Simple decision tree structure for predicting external corrosion rate of carbon steel in soil.

As illustrated in Fig. 10, the essential components of a decision tree model are nodes and branches, and the most significant steps in model construction are splitting, stopping, and pruning. There are three basic types of nodes: root nodes, internal nodes, and leaf nodes. The root node, also known as the decision node, represents a choice that will result in the splitting of data into two or more subsets by branches, as multiple opportunities arise. Internal nodes often called opportunity nodes, reflect the various options available in the tree structure. The result is represented by a leaf node, which is also known as the end node. The tree begins with the root node, which contains all the data, and then divides the nodes into various branches using intelligent strategies.

In addition to the structural composition of the tree, there are steps to build the models which include splitting, stopping, and pruning. When creating a model, the most essential input variables should be defined first, and then the records at the root node and subsequent inner nodes should be divided into two or more categories or buckets based on the state of these variables. This separation technique is repeated until the halting or homogeneity conditions are reached. In most circumstances, not all possible input variables will be used to construct the decision tree model. In some cases, a single input variable will be used many times at different levels of the decision tree. A different algorithm was written to assemble a decision tree, and this can be utilized in the problem. Some of the common tree decision algorithms are classification and regression trees (CART), iterative dichotomiser 3 (ID3), C4.5, and Chi-square automatic interaction detector (CHAID). CHAID was used in this study.

The Chi-square automatic interaction detector (CHAID) is an algorithm that generates a decision tree using Chi-square statistics to determine the optimal decomposition. Continuous predictors are divided into categories with an approximately equal number of observations, whereas categorical predictors are divided into categories with an approximately equal number of observations. For each category predictor, CHAID performs all potential cross-tabulation until the best result is obtained and no further splitting is possible. The CHAID approach can be used to visualize the relationships between the split variables and the accompanying related factor within the tree.

**Figure 11:**
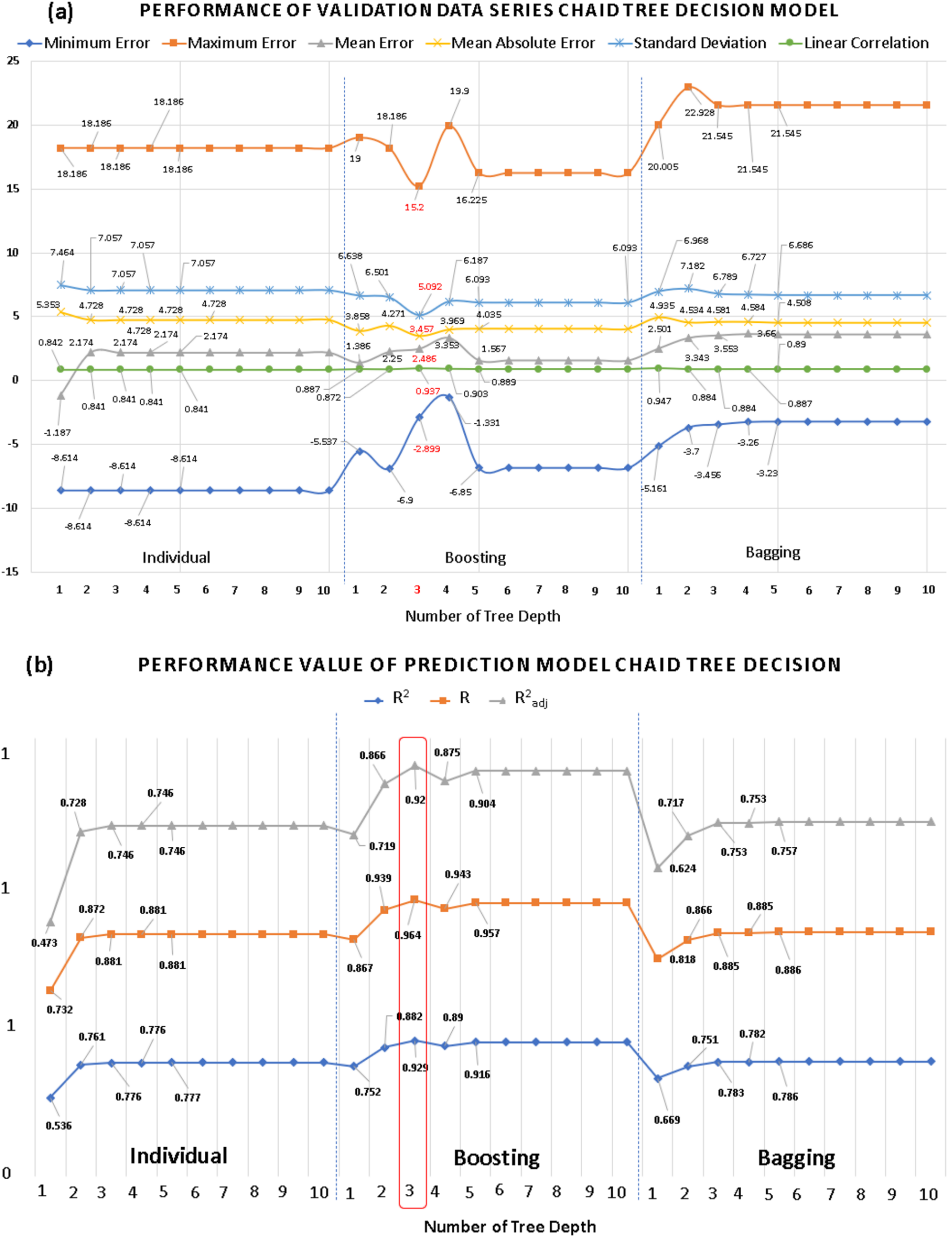
Performance evaluation and comparison (**a**) validation data (**b**) prediction models of the single, boosting and bagging models of CHAID decision tree for modeling *i*_corr_ of carbon steel in soil with variations in the number of tree depths.

Three methods of standard and homogeneous ensemble learning (bagging, boosting) were employed with the different numbers of tree depths. From the maximum tree depth to the value 5 and onward, the predicted values were identical, and the number of tree depths did not grow until value 5. Table 4 summarizes the results of the evaluation of the 3 models above with minimum error, maximum error, mean error, mean absolute error, standard deviation, and linear correlation value. The prediction results from the boosting method with tree depth of 3 had the lowest MAE value and all other parameters are the best of the validation dataset in Fig. 11a. Even the model CHAID tree decision with tree depth of 3 has the most R, R^2^, R^2^_adjusted_ values in Fig. 11b. Therefore, the boosting ensemble learning method with a tree depth of 3 was chosen as the optimal model for CHAID tree decision.

**Figure 12:**
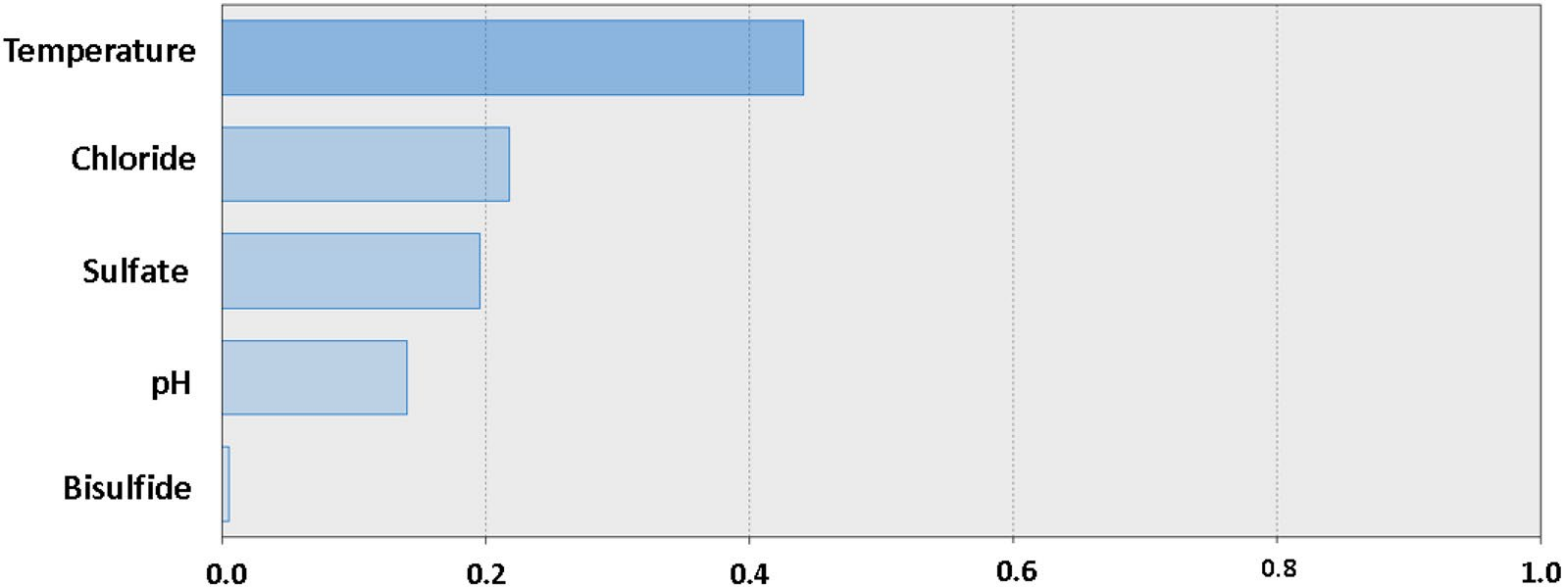
Chart for the standardized effects of temperature, chloride, sulfate, bisulfide, and pH, as predicted by CHAID Tree Decision with three tree depths on the corrosion current density of carbon steel in soil.

Regarding the evaluation of the sensitivity of the CHAID tree decision model to the factors, temperature continues to be the dominant factor affecting the rate of soil corrosion. Chloride and sulfate ranked second while pH and bisulfate remained the two lowest influencing factors as shown in Fig. 12.

### Linear regression algorithms

The final algorithm applied in this study is linear regression (LR). Simple linear regression has one predictor variable (X) used to model the response variable (Y). But in this case, the response variable of corrosion current density of carbon steel was affected by more than one predictor variable. Therefore, the multiple linear regression algorithm must be used. Multiple linear regression algorithms are a method to study the relationship between many predictor variables and one response variable. It is often used for prediction in machine learning used for supervised learning. Based on the given data points, it tries to plot a line that models the best points, and its main objective in this algorithm is to find the best fit line. The general formula of the multiple linear regression model is:

1 $$Y={\beta }_{0}+\sum_{i=1}^{n}{\beta }_{i}{X}_{i}+\varepsilon$$

In this study, Y (corrosion current density) = output/response variable, $\beta =$ coefficients of the model. X_1_ (temperature), X_2_ (chloride), X_3_ (pH), X_4_ (sulfate) and X_5_ (bisulfide) are the independent variables. After putting labeled data into the software, an equation was obtained:

2 $$Y=0.036 \, pH+0.489\, Chloride+0.078\, Bisulfide-0.179\, Sulfate+0.652\, Temperature$$

Because linear regression algorithms use data to fit the equation, a random amount of the same dataset still produces the same equation, and homogeneous ensemble learning is not effective in this algorithm. The accuracy of the equation is listed in Table 3.

**Table 3:** Summary of linear regression model for predicting *i*_corr_ of carbon steel in soil.

R	R square	Adjusted R square	Std. error of the estimate
0.928^a^	0.861	0.842	4.347795

The R-value represents the correlation, and it was 92.8%, indicating a very high degree of correlation. The R^2^ value indicates the percentage of the total variation in the response variables.

As shown in Table 4, ANOVA reports the fit of the regression equation to the data, it shows that the regression model predicts the response variable well. The regression row and the Sig column show the statistical significance of the run regression model. Here, p < 0.05, indicates that the regression model predicts statistical significance on the response variables (it fits the data). To confirm the statistical significance of the fit of the overall regression model, the obtained F-value was compared with the F-critical value. The F-critical value in the F-distribution was determined by the cut-off between columns degrees of freedom (df) of F numerator and df of the denominator or error of F.

- df of F numerator = number of beta parameters in the regression model—1 = 6 − 1 = 5
- df of F denominator = n—number of beta parameters in the regression model = 43 − 6 = 37.

**Table 4:** ANOVA of linear regression model for predicting *i*_corr_ of carbon steel in soil.

Model	Sum of squares	df	Mean square	F	Sig
1. Regression	4329.938	5	865.988	45.811	0.000^b^
2. Residual	699.423	37	18.903		
3. Total	5029.361	42			

Looking up in the 5% distribution, the F-critical value (df of F numerator, df of F denominator) was from 2.534 to 2.450. The result of the F-test total panel ANOVA was 45.881, which is much higher than F-critical. This indicates that the overall regression model was statistically significant, and variables of pH, chloride, bisulfate, sulfate, and temperature are significant predictors of the response variable of the corrosion rate.

Table 5 provides the information needed to predict the corrosion rate from the 5 factors as well as to determine if these 5 explanatory variables contribute in a statistically significant manner to the model by looking at the Sig. column. In the Table 5, the results show that there are 3 coefficients of chloride, temperature, and sulfate that are statistically significant (p < 0.05). Furthermore, the values in column B can be used in the unstandardized coefficients column. However, since the values in this study have different units, it is most appropriate to use standardized coefficients. The important result MAE of linear regression is 3.696, which is a pretty good value.

**Table 5:** Coefficients of linear regression model for predicting *i*_corr_ of carbon steel in soil.

Model	Unstandardized coefficients		Standardized coefficients	T	Sig
	B	Std. error	Beta		
Constant	− 0.360	4.172	–	− 0.086	0.932
pH	0.222	0.481	0.036	0.461	0.648
Chloride	0.004	0.001	0.489	7.838	0.000
Bisulfide	8.916E−5	0.000	0.078	1.175	0.248
Sulfate	− 0.009	0.004	− 0.179	− 2.242	0.031
Temp	0.366	0.038	0.652	9.597	0.000

**Figure 13:**
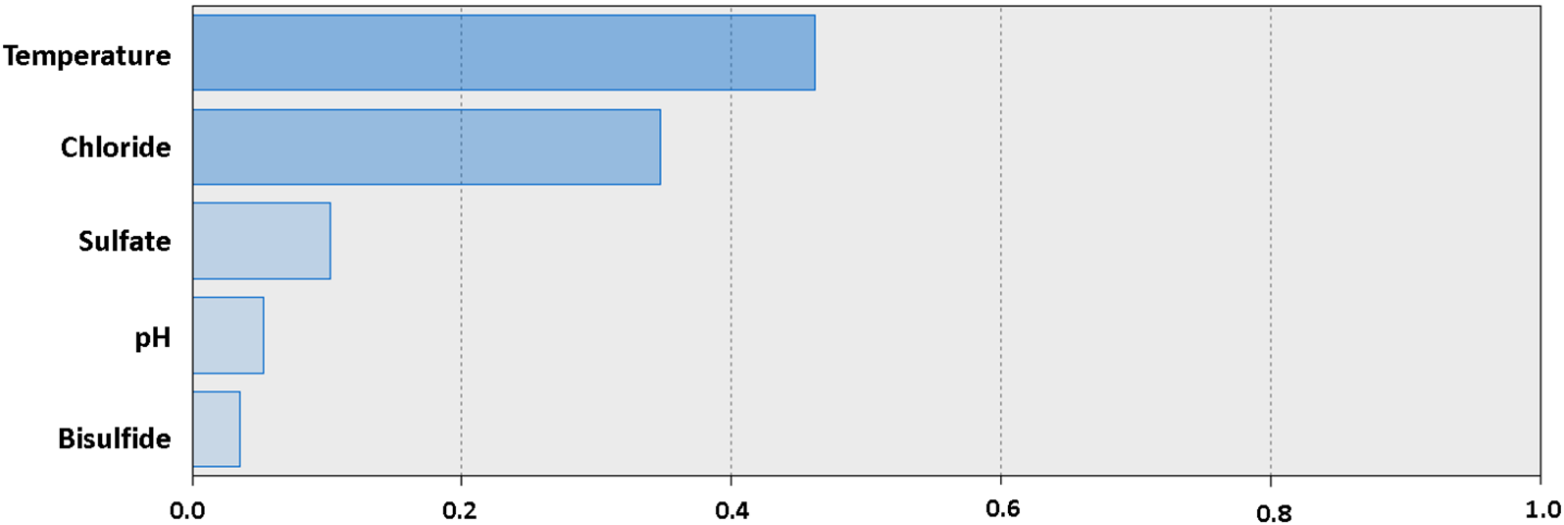
Chart for the standardized effects of temperature, chloride, sulfate, bisulfide, and pH, as predicted by LR on the corrosion current density of carbon steel in soil.

Evaluating the influence of the research factors in LR, temperature is still the factor that the corrosion rate of carbon steel is most sensitive. In Fig. 13, chloride increases the degree of influence on the corrosion rate of carbon steel higher than the 2 algorithms above, and the remaining 3 factors are still considered to have a minor influence on corrosion rate.

#### Heterogeneous ensemble learning—stacking

All three individual algorithms and homogeneous algorithms in this study are simple and easy to implement and the prediction results are quite accurate, however, the heterogeneous ensemble learning method was applied in this section to improve the accuracy as much as possible.

**Figure 14:**
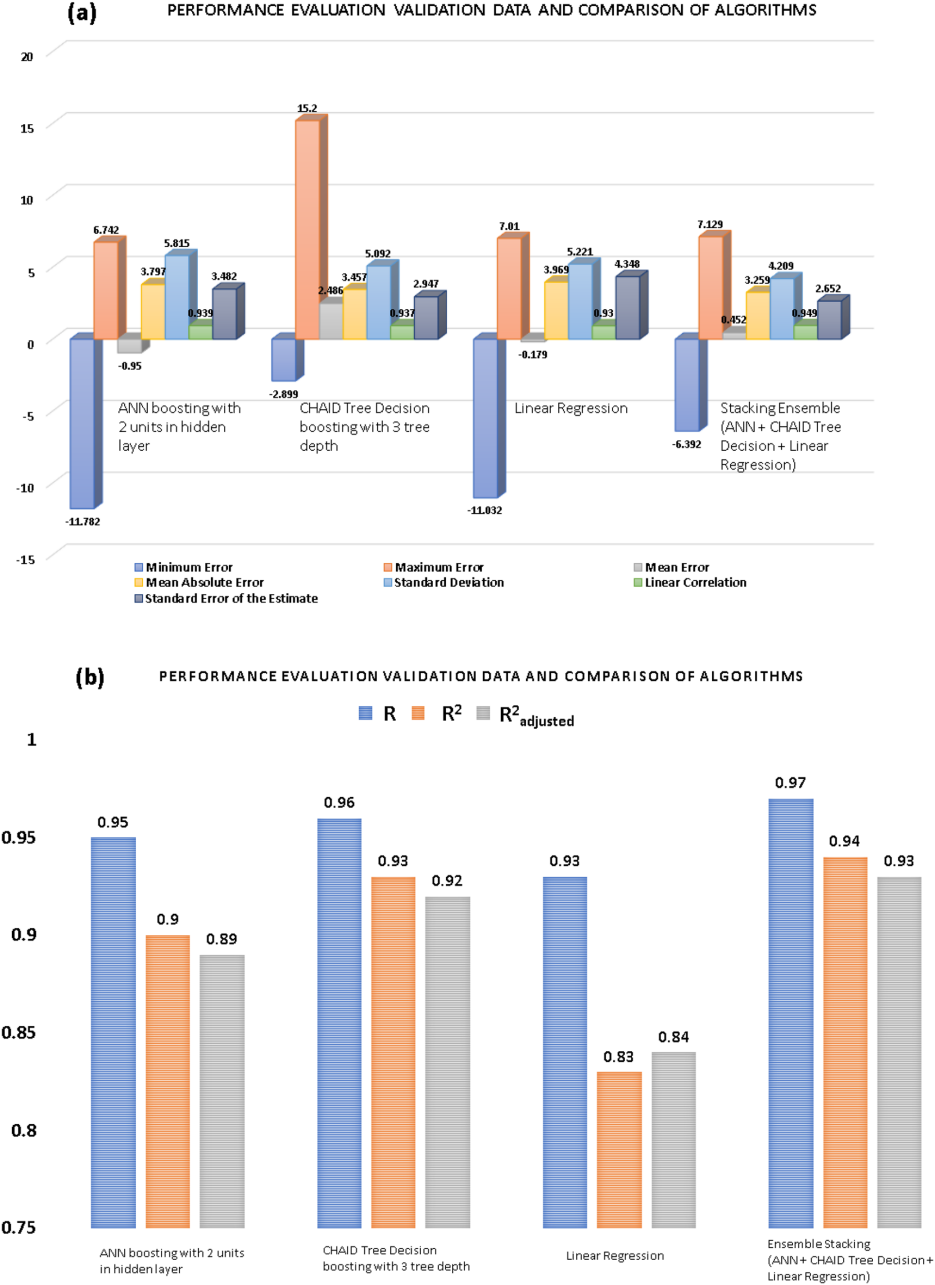
Performance evaluation and comparison (**a**) validation data (**b**) model of methods.

**Figure 15:**
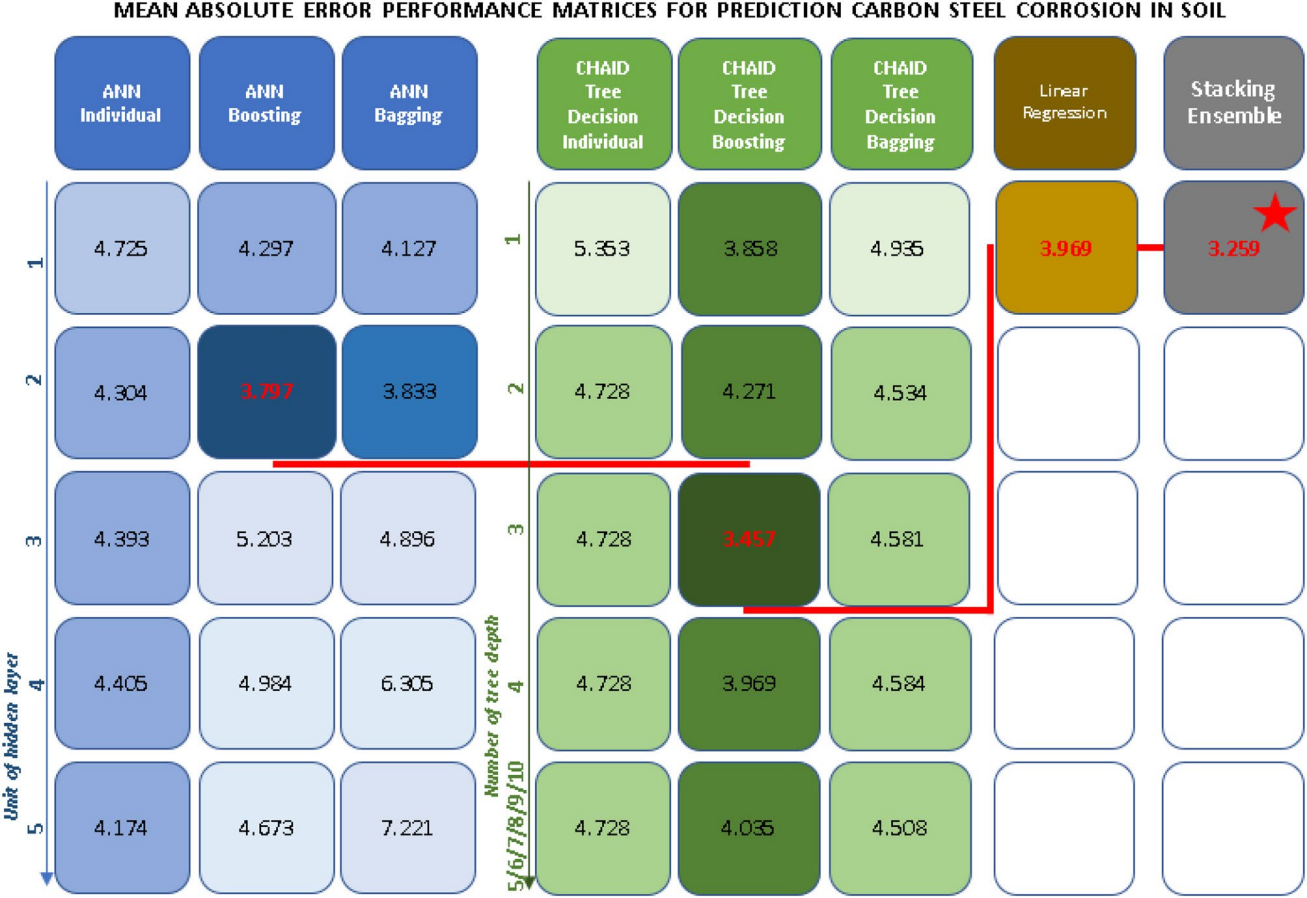
Mean absolute error performance matrices for prediction carbon steel corrosion in soil.

**Figure 16:**
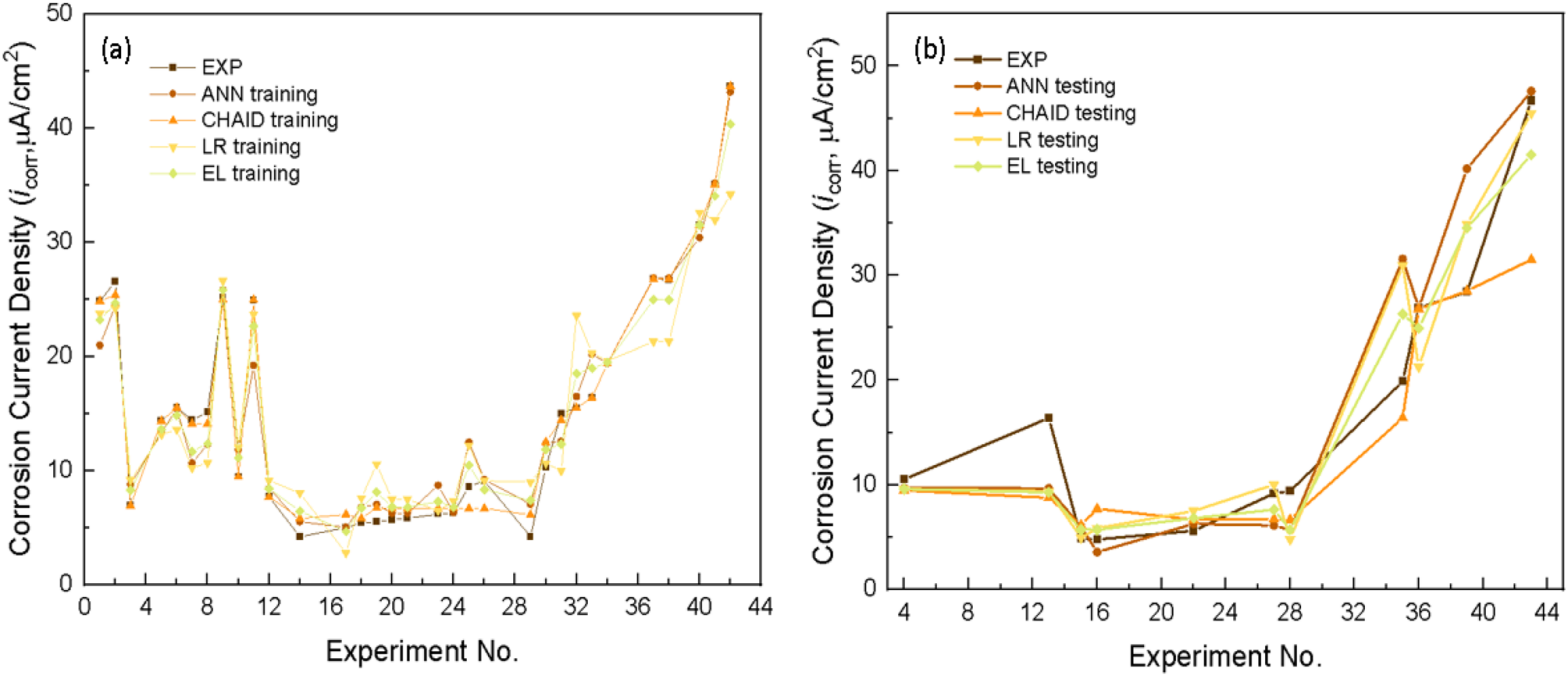
Predicted value versus the measured external corrosion rate (**a**) training data (**b**) testing data of carbon steel in soil.

According to Fig. 14a, the boosting method of ANN with 2 units in the hidden layer gives the best result with the MAE of 3.797. Boosting CHAID with a tree depth of 3 gives the best results in the CHAID decision tree algorithm with the MAE of 3.457, and the prediction results of the linear regression showed the MAE of 3.696. In comparing the three algorithms, it seems that the CHAID decision tree gave the best prediction results. However, heterogeneous ensemble learning was implemented for a better predictive value to improve the model. Indeed, the three best-selected models above combined for a model with the MAE value of 3.259, which is the smallest value of all the models running in this study, all other values, the stacking ensemble method is still the model with the most accuracy even in Fig. 14b and summary MAE performance matrices in Fig. 15. The R, R^2^, R^2^_adjusted_ values of the stacking ensemble method are still the best model. The training results of the four models and checking the prediction results with the validation dataset are shown in Fig. 16.

**Figure 17:**
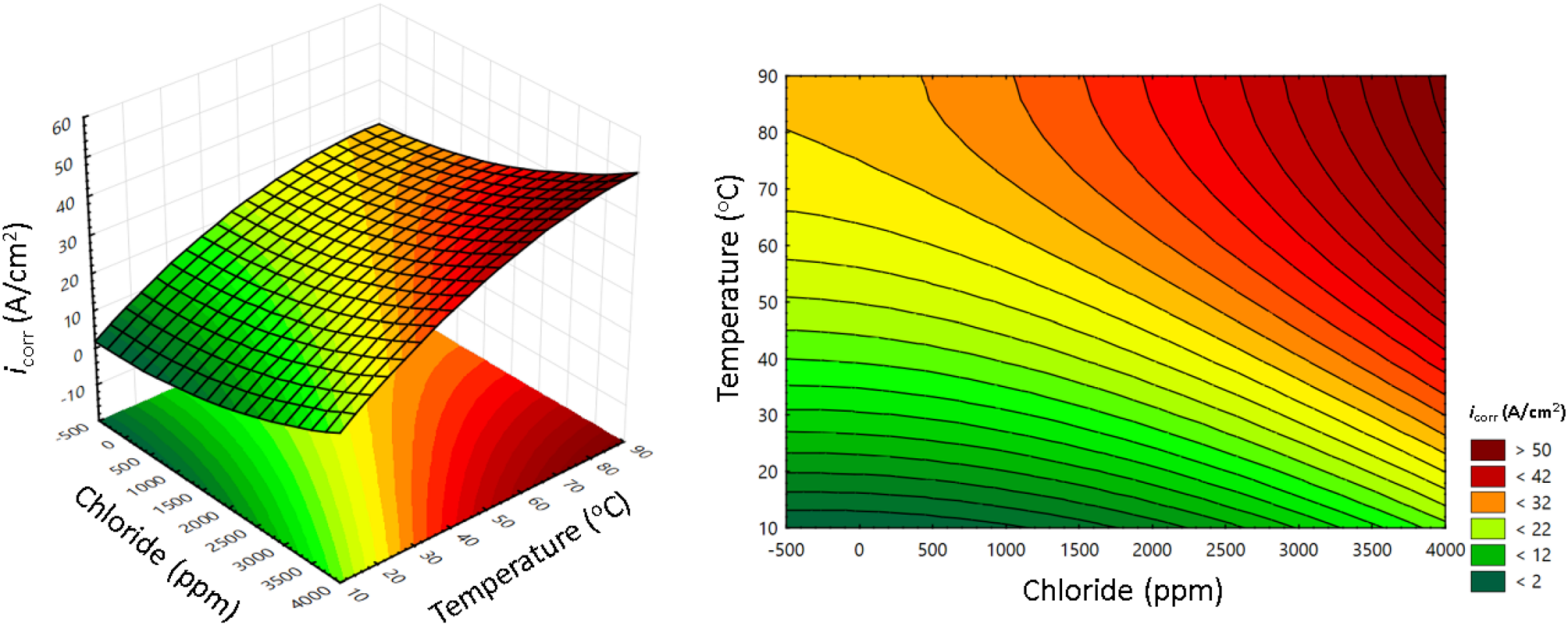
Influence of two major factors (temperature, chloride) on the corrosion rate of carbon steel in soil.

Finally, the two major factors that the corrosion rate of carbon steel in soil temperature and chloride are shown on the response surface and contour surface according to the model of the stacking ensemble method in Fig. 17.

## Conclusion and future work

The purpose of this paper was to build an optimized model to predict the external corrosion rate of carbon steel canisters in soil. After performing a series of models to find the most accurate model, the ensemble learning stacking method was identified as the optimal method to predict behavior using the studied dataset. Although the method showed excellent accuracy on the predicted values in this dataset for predicting the rate of corrosion outside the pipeline, that does not mean that this method is the best in all cases. Therefore, a number of models must be evaluated for each different dataset to find the optimal model. To improve the reliability of the model, it is necessary to provide a large amount of data on corrosion soil and provide more predictors that affect soil corrosion such as moisture, porous. Besides applying the ML, the significance of the input variables was also determined through sensitivity analysis, and the corrosion rate of carbon steel are the most sensitive to the temperature and chloride.

## Data Availability

The authors confirm that the data supporting the findings of this study are available within the article. Raw data that support the findings of this study are available from the corresponding author, upon reasonable request.
